# Towards System Changes: Experiences of General Practitioners and Nurses in Facilitating Appointments and Referrals for Africans from Refugee Backgrounds in Australia

**DOI:** 10.1002/hpja.70218

**Published:** 2026-07-28

**Authors:** Prince Peprah, Emmanuel Brenyah Adomako, Dipti Zachariah, David Ajak Ajang

**Affiliations:** ^1^ Australian Institute of Health Innovation, Macquarie University Sydney Australia; ^2^ International Centre for Future Health Systems University of New South Wales Sydney Australia; ^3^ Department of Social Work, School of Social Sciences University of Wollongong Wollongong Australia; ^4^ Patient and Carer Experience, Clinical Governance Western Sydney Local Health District, NSW Health Sydney Australia; ^5^ Sydney Health Literacy Lab, Sydney School of Public Health, Faculty of Medicine and Health The University of Sydney Sydney New South Wales Australia; ^6^ Independent Research Consultant Perth Australia

**Keywords:** access, Australia, challenges, GPs, models of care, primary healthcare, refugee background, specialist care

## Abstract

**Objective:**

This study explored the experiences of general practitioners (GPs) and nurses working in specialised refugee health services when facilitating specialist hospital appointments and referrals for Africans from refugee backgrounds in Australia.

**Methods:**

An exploratory‐descriptive, qualitative study was conducted. Purposive sampling was used to recruit 12 GPs and nurses across three states: New South Wales, Victoria, and Queensland. Semi‐structured interviews were conducted and recorded via Zoom. Reflexive thematic analysis was undertaken to generate themes. This manuscript is based on the Consolidated Criteria for Reporting Qualitative Research (COREQ).

**Results:**

This study revealed two analytical constructs. The first construct explains the various challenges that GPs and nurses working in specialised refugee health services face in facilitating referrals and appointments for their patients including: (a) assumptions in communication; (b) use of abbreviations in appointment reminders; (c) navigating cultural and religious sensitivities in appointments; (d) difficulty getting clinicians to use interpreters; and (e) silos working. Nevertheless, the second construct explains that the providers in this study remained passionate and committed to helping refugees access the care they need.

**Conclusions:**

It is essential that primary healthcare professionals in specialised refugee services feel supported, encouraged and empowered to enable access to appropriate specialist hospital services by refugees, thereby preventing unwanted health outcomes.

**So What?:**

This study reveals the needed changes to inform equity‐based policies and system level interventions that promote timely access to specialist services for refugees to improve health outcomes.

## 1 Introduction

Migration and access to healthcare services are critical determinants of health and well‐being, especially for refugees [1, 2, 3]. Studies across different countries indicate that refugees have limited access to comprehensive and essential health services [4, 5, 6]. Globally, refugees' limited access to health services is influenced by multiple individual, institutional and systemic factors. Factors such as lack of awareness of healthcare rights and entitlements, sociocultural factors, communication and linguistic barriers, mistrust of interpreters, and limited health literacy play an essential role in refugees' access to health services [4, 7, 8, 9]. In some cases where free universal health services are not available, refugees are unable to afford medical consultation fees, transportation to appointments and pharmaceutical and other health‐related expenses [10]. In Australia, newly arrived refugees are found to be unfamiliar with the local environment and healthcare system, which serve as barriers to accessing health services [9, 11, 12].

In Australia, specialised refugee health services with specialised skills and experience have been set up. These services are established to address the intricate health challenges and obstacles to accessing mainstream healthcare encountered by refugees, as well as the difficulties faced by many private practice general practitioners (GPs) in providing adequate care to refugees [13, 14]. Their core responsibilities include conducting initial comprehensive health assessments of newly arrived refugees and arranging long‐term care, including appropriate specialist hospital services [15, 16]. In addition, they provide care upon referral from generalist refugee‐focused health services and mainstream primary or secondary care [17]. Examples of specialised refugee health services in Australia include the New South Wales Refugee Health Services, torture and trauma services and refugee paediatric clinics. Other not‐for‐profit agencies and services in different States and Territories, such as the NSW Service for the Treatment and Rehabilitation of Torture and Trauma Survivors, Program of Assistance for Survivors of Torture and Trauma and other specialist mental health supports within Local Health Districts, also provide specialised services for the treatment of torture and trauma [18].

Evidence shows that many refugees and people from refugee‐like backgrounds, such as asylum seekers, need specialist hospital appointments and referrals for various conditions and diseases, such as heart, skin, mental health, children's health and joint and muscle disorders, after going through initial health screenings and assessments [19, 20]. One important function of health professionals within specialised refugee health services is making and facilitating these referrals and appointments for newly and recently arrived refugees to ensure ongoing care [13, 21]. Some studies have generally explored the challenges that nurses and GPs face in providing care for refugees, mainly at the primary care level in Australia [22, 23, 24] and beyond [25, 26, 27, 28].

However, few studies have also focused on experiences of specialised refugee health nurses in Australia [29, 30]. There is a lack of studies that specifically examine the experiences of specialised refugee care providers (including both GPs and nurses) in booking and facilitating specialist hospital appointments and referrals for refugees. Specifically, the system and service‐level challenges that health professionals working in specialised refugee health services face when facilitating hospital specialist care for newly arrived refugees remain largely unknown, especially in Australia. This knowledge is essential to promote policies and practices that ensure that refugees have timely access to appropriate specialist care upon arrival. To that end, this qualitative study explored the experiences of GPs and nurses in specialised refugee health services when facilitating specialist hospital care (appointments and referrals) for refugees in Australia.

## 2 Methods

### 2.1 Research Setting

This study was conducted in three Australian states: New South Wales, Victoria and Queensland. These states have the highest refugee resettlement, with Victoria receiving around one‐third of all refugees and people seeking asylum in Australia [31].

### 2.2 Approach, Sample and Recruitment Procedure

This study adopted the exploratory‐descriptive qualitative (EDQ) design [32] because little research has investigated the experiences of primary healthcare professionals when facilitating specialist hospital referrals and appointments for refugees, particularly those from African countries, in Australia. This paper is derived from a larger study assessing the responsiveness of primary healthcare services and professionals to African refugees in Australia. Details about the methods have been reported elsewhere [33]. Data from twelve (12) healthcare professionals from New South Wales, Queensland and Victoria, including GPs (*n* = 4), registered nurses (*n* = 5), nurse practitioners (*n* = 2) and paediatricians (*n* = 1), who were working within specialised refugee health services. Although 14 healthcare professionals were recruited for the larger study, 12 provided data/information related to the topic discussed in this study and were therefore included (see Table 1 for background characteristics of the participants), are reported in this paper. Only background and demographic information collected during the interview has been reported.

**TABLE 1:** Characteristics of the participants (*n* = 12).

Gender	
Male	4
Female	8
Age (years)	
30–39	4
40–49	5
50 and above	3
Discipline	
General Practitioner	4
Nurse Practitioner	2
Registered Nurse	5
Paediatrician	1
Duration of practice	
10–15	3
16–20	2
20–25	4
Above 25	3
State	
New South Wales	6
Victoria	3
Queensland	3

They were eligible to participate in this study if they were directly involved in providing primary care (consulting, diagnosis, prescribing and dispensing medication) and/or in giving health behaviour advice and counselling to refugees. The study flyer was sent to health services and professional bodies working with people from refugee backgrounds. These services and organisations shared the flyers with health professionals who provide primary healthcare to Africans from refugee backgrounds. Those who received the invitations were followed up via email, text message or phone to provide further details on the study and interview process and to arrange an interview date if the participant was interested.

### 2.3 Data Collection

The first author (PP) conducted semi‐structured interviews with the participants between March and August 2022. PP is an experienced qualitative researcher and interviewer, with a good understanding of the Australian healthcare systems and services. A semi‐structured interview guide was developed and refined throughout the interviewing process to gather further developing themes and concepts. The interviews lasted 1 hour on average and were conducted in English using an institutional Zoom platform. With the participants' written consent, all interviews were digitally recorded and transcribed verbatim.

### 2.4 Data Analysis

A reflexive thematic analysis guided the data analysis [34]. All the data were managed in NVivo (version 12). Data familiarisation occurred via multiple readings of the transcripts. PP read all the transcripts, whilst the other authors read different subsets to guide the development of the coding framework. As an iterative process, data analysis involved both inductive (data‐driven) and deductive (researcher‐driven) approaches, in line with the research question(s), to generate a coding framework that reflects emerging patterns and themes. This data analysis provided an opportunity to triangulate the data with existing findings [24]. The authors discussed the relationships between categories to develop and identify themes. The refined coding framework was applied to all the remaining transcripts and important emerging themes were merged and checked across the full coded dataset. The reporting of this qualitative study follows the consolidated criteria for reporting qualitative research (COREQ) [35].

## 3 Findings

The thematic findings describing primary healthcare professionals' experiences of facilitating specialist hospital appointments for refugees are reflected across two analytical constructs: systemic and service‐level challenges in facilitating appointments and referrals and maintaining passion and commitment amid these challenges.

### 3.1 Systemic and Service ‐Level Challenges in Facilitating Appointments and Referrals

This construct is related to systemic and service‐level challenges that primary healthcare professionals faced in facilitating appointments and referrals for their refugee patients. These challenges included assumptions in communication regarding appointments; use of abbreviations in appointment messages; difficulty getting specialists to use interpreters; navigating cultural and religious sensitivities in appointments; and silos working.

#### 3.1.1 Assumptions in Communication

Participants reported that poor communication by hospitals and specialist services was a key challenge primary healthcare professionals had to deal with. Participants noted that hospitals and specialist services often make assumptions in their communication. It was mentioned that hospitals and specialist services assumed that all refugee patients can read English, so messages such as appointment reminders and letters are often written in English.

You know, outpatient services, make assumptions about people's knowledge and understanding of information that they send. They all need to lift their game a little bit in how they communicate. I think we also sometimes don't pay attention to the language that people speak. So, we have health systems and data sets and alerts that say, you know, somebody speaks Dinka, or somebody speaks whatever, like Arabic and yet sometimes the administrative people involved in those positions don't necessarily pay attention to that and don't realise, well, maybe I shouldn't send something in English because they won't understand. Maybe I should get an interpreter and ring them, you know, because either they don't notice it or they don't care or they know it will be more work for them to provide that level of communication. (GP 2)

Some of the providers mentioned that, in most cases, text messages sent to refugee patients do not contain key information, such as the type of appointment and which family member the appointment is for. It was mentioned that many of the refugee patients have large families and it is hard for them to identify the patient the appointment is for when reminder text messages fail to mention who the appointment is for.

I have had clients come here to my community health, and they show me the text message on their phone, for example, it might have been a certain hospital without stating the time and the appointment reason. They didn't tell them what the appointment was: was it a surgical appointment, an orthopaedic appointment, a cardiology appointment, or an appointment for one of the kids? A lot of these families may have from six to 10 children. (Nurse 8)

The participants described situations in which refugee patients returned to them seeking more details about their appointments, as an extra burden on their work, as they needed to spend several hours looking up information for their clients.

#### 3.1.2 Use of Abbreviations/Short Hands in Appointment Messages

Using abbreviations in text messages by specialist hospital services was another challenge that primary healthcare professionals faced in facilitating access to health services for refugees. Almost all participants mentioned that using abbreviations in text messages for refugee patients is poor practice in healthcare. Yet, many hospitals continue to do so when communicating appointment information to refugees.

We think that using abbreviations is a bad practice in health, and you shouldn't do it, particularly for people who don't speak English, for them to understand what the abbreviation means… is a bit naive. So, I think, I think the health service should not do it, but unfortunately, they do it often. (GP 1)

For instance, some participants mentioned that service providers often used abbreviations that are hardly known to newly arrived refugees in text messages, leading to missed appointments.

Communication has always been the issue…and for communication, I mean how hospitals and specialist services communicate with refugee patients about their appointments. So, we have had families, for example, in … who were sent to a hospital, but they couldn't go because they didn't understand the message. You know, they received a text message about their appointment, but it didn't include the location. It just said they had an appointment with a certain doctor at CHW. In the first place, what does CHW mean? CHW stands for Children's Hospital at…, but his family lived in …, so they didn't know what CHW stood for. They didn't turn up to the appointment because they didn't know where to go. They didn't know who to call. I mean, this is a good example. (Nurse 5)

Another participant added a similar situation in which refugee patients received messages with abbreviations, leading to confusion and missed appointments.

There was another gentleman who had been sent a text. He had a neurological appointment that had been made six months ago, and it was for …, but he didn't know what JHH meant. So, you know they're just one of the many challenges that we deal with. (Nurse 10)

Most of the nurses mentioned that, since many refugees know where to find them, the responsibility often falls on them to look up more details about their appointments.

… things become complex, so they need more support… they keep bouncing back because they know where to find me and they know that I will never say no. I will always help or try my best to find someone to support them (Nurse 7)

#### 3.1.3 Navigating Cultural and Religious Sensitivities in Appointments

Participants, especially GPs, mentioned that it is often a difficult task when making referrals for refugee patients, particularly those from culturally diverse backgrounds like Africans, due to cultural and religious issues. These GPs mentioned that some refugees have preferences for male and female providers and in some cases, their preferred providers might not be available.

… like for example, some males don't want to see a female doctor. Similarly, some females do not want to see a male doctor due to their cultural and religious beliefs, especially those from Africa and the Middle East. But when making referrals becomes a problem for you, the doctor, especially in cases where their preferred clinicians are not available. Sometimes you need to spend time explaining to them to see if they would like to see any available doctor, but that is not an easy task. It is not an easy conversation at all… (GP 2)

This GP shared that some refugees often return and complain to them about how they felt the providers they saw were culturally insensitive. They also mentioned that some refugees refuse to see the providers again.

Okay, sometimes, like if you send them to a specialist who might not be from that background, they will not be aware of the cultural background. The patient might return unhappy. They refuse to go back to that doctor, not because he is not knowledgeable or does not treat them, but maybe because he wasn't aware of the cultural issues, and these include religious issues. So, it is always an issue for us when you are referring to them… you must be careful. So, I usually ask my clients to please, when you call to make an appointment, ask for a female or male technician on that day, to make sure what you like is booked for you on the day of the appointment. (GP 2)

Some nurses mentioned that they mostly had to accompany their clients to specialist appointments upon their clients' request. This is because their presence makes them feel comfortable and safe. However, following clients to appointments often overburdens them.

I always hear our clients say I wish you would go to the appointment with us. They will say, I think the doctor will respect my culture when you are there, so I feel okay. I accompany a lot of them to appointments, but it is an extra work that sometimes overburdens us. (Nurse 2)

#### 3.1.4 Difficulty Getting Clinicians to Use Interpreters

Another critical challenge in communication was encouraging clinicians to use healthcare interpreters. Participants felt that some providers within specialist hospital services are on some occasions reluctant to use interpreters due to limited consultation time and inadequate knowledge of interpreting. Participants described getting health professionals within specialist hospital services to use interpreters as one of the most significant problems that they face when facilitating access to health services for refugees.

Also, as I said, a lot of the doctors out there don't like using interpreters, and they do the wrong thing and use family members, or they don't use an interpreter at all, and things get lost in translation. Getting them to use interpreters is one of the biggest problems, and you know, getting them to take their time so people can understand things better is another thing. I think it is because of time: within 5 minutes, you should leave the office so someone can come in. I also think it is because of the system and structures we have. So, things don't get followed up. (Nurse 3)

Participants mentioned that many refugee patients often return to their clinic when they are unable to attend their appointments due to providers' unwillingness to use interpreters. Participants further shared that they frequently advocate or convince specialists to use interpreters with refugee clients who are not able to speak English. They mentioned that such advocacies often delay refugees' care‐seeking.

We had people come back to our clinic who had not used interpreters, which mostly put pressure on us. We often need to convince such doctors to accept our clients. Sometimes we need to change doctors, which delays care for our clients. It is a big challenge for us. (GP 3)

#### 3.1.5 Silos Working

Participants discussed the need to connect with other services and facilitate referrals to them to promote timely access for refugees. Despite the importance of connection and integrated care, it was revealed that many participants struggled with trying to refer refugee patients to the broader range of social, community and health services that refugees required. Many participants mentioned a disconnect between services and verbalised feelings of professional isolation (working in silos) in care coordination across the primary, secondary and tertiary care interfaces.

We need to work together to help our clients, but unfortunately, health works in a lot of silos… You know mental health is here, surgery is here, cardiac and medical support is here. So, particularly with clients that may, for example, require comprehensive care, it's tough to pull all those strings together so that we're looking at a person from a whole perspective, not just health condition solving. (Nurse 4)

We work in silos. So that's a big challenge… it is just the coordination of it all really. (Nurse 6)

### 3.2 Maintaining Passion and Commitment Amid Challenges

The second construct demonstrates the passion, commitment and sense of moral responsibility that participants attach to facilitating access to care and appropriate services for refugees, despite existing service and system‐level challenges. Despite the challenges encountered in enabling access to healthcare services for refugees, almost all the healthcare professionals in this study reported that their source of strength and resilience is the passion and love they have for caring for refugees. The healthcare professionals expressed their willingness to take on additional tasks to ensure that refugees have access to the necessary healthcare and other social services. They found facilitating access to healthcare services for refugees as rewarding, humbling and enjoyable:

It is really, really interesting… I find it personally interesting. Quite often, I really feel very humble. [I think about] what people have experienced, and you know… the stories and their travels of escaping torture and trauma. So, on a personal level, I found it really interesting, and I like the work on a professional basis. (Nurse 10)

All the healthcare professionals expressed happiness and satisfaction with the impact they make on the lives of refugees. Some healthcare professionals narrated how happy they become when they see the clients they assist making good progress in their lives beyond healthcare.

I'm a GP [and I] have been a GP for 30 years or so. I see mostly refugee patients, but they're more assimilated into the community. I enjoy that sort of work… I particularly enjoy watching people grow in health literacy and in self‐management skills, let alone start to get jobs and learning… [it is] exciting to see that happen. (GP 3)

One nurse who conducts initial refugee home visits also comprehensively captured the pride and joy of meeting refugees in their homes on arrival and of assisting them to access healthcare and other social services, as well as to settle into a new environment.

Oh my gosh, it's quite a privilege, actually, and I think it is because we first meet people when they arrive… we go to their home [to] visit them, and that's pretty special because we get to see them in their holistic family environment. You know we're fortunate because they don't know us… so it's a privilege, you know, and I love the families that we meet… we give them the information about health services and systems here and try and link them to services. (Nurse 2)

Some of the healthcare professionals saw facilitating access to healthcare and other social services, as well as helping refugees start a new life in Australia, as an exceptional opportunity for them to contribute to fulfilling humanitarian values and principles underpinning refugee healthcare. One nurse narrated how gratifying it was for her to have assisted a refugee couple in becoming pregnant.

I assisted an African couple with [In vitro fertilisation] (IVF)… because the husband has been tortured, [he] was unable to help his wife get pregnant, and that was a success for them to have a baby… it was gratifying, and it is all about humanity. (Nurse 5)

Healthcare professionals' willingness to help and make things work for refugees in the face of challenges was also driven by a sense of moral commitment and responsibility, equity and human rights. The participants felt that health is a fundamental human right and that everyone, including refugees, has the right to access appropriate healthcare. They mentioned that many of the refugees have experienced significant access to care. Thus, helping them to access services and settle successfully in Australia promotes equity.

I have a fundamental view that it's the right thing to do. If people have been treated so appallingly in their country, then we ought to share some of our riches with them. So, I should share the opportunity I've had to have a decent education with these people and help them, you know, that's our job as doctors. However, I think it's just something that I really believe that everyone is equal, no matter what their colour, their skin, their language or their background, and they all deserve good healthcare. (GP 1)

Another participant also added to the above comment by emphasising personal conviction and ethics:

Again, personal conviction and ethics also drives me, and I think and other colleagues as well to make sure that these people who have suffered a lot of trauma and challenges understand our complicated system and also be able to understand what is going on and happening to their system, how to book appointment and walk to the appointment and understand medications and other important information. (GP 2)

The participant further discussed how the need to promote equity and fairness inspires them to help refugees access health services and successfully settle in Australia.

I think the last thing is equity, the need for equity, because we are privileged to understand the system, book an appointment, interact well with providers and as a result we have better health outcomes. So, to ensure fairness and equity, we need to let people who, for instance never been to school or had their education interrupted for wicked reasons and speak different language understand what we have here to be also able to access services that bring their health to a meaningful level. (GP 3)

Some healthcare professionals, however, showed frustration, even with personal commitment and conviction, that refugees need health services.

Just trying to defer an appointment today with the neurological team in … how the hell do they think the average person can do it, let alone someone with limited English. No wonder so many appointments fall over and are not economically viable. (Nurse 4)

Others also raised doubts about their ability to sustain their efforts and commitments to promoting access to services for refugees in the long run if service‐ and system‐level challenges persist and become overwhelming.

For now, we are doing great, and our shared commitment and passion sustain us, but we cannot tell what may happen in the future if the challenges in services and systems are not addressed for refugees to access services easily. (Nurse 8)

## 4 Discussion

Practical, clear and culturally appropriate communication is crucial in healthcare, especially among the refugee population, as it promotes trust in information and services. However, several assumptions and perceived bad practices were made in the communication of hospital and specialist appointments. These assumptions and bad practices included the use of abbreviations/short forms in appointment and reminder messages, limited details about appointments and the use of English as the only medium for appointment communication. Assumptions in health communication, particularly in refugee health, have been previously argued [9, 36]. It was clear from our study that hospital and specialist services incorrectly assumed that all refugee patients could read and understand English and were also familiar with abbreviations for popular hospital names.

Peprah et al. [36] in a study of negative experiences in accessing health services among African refugees reported similar findings regarding the inherent assumptions healthcare providers make about refugees. In line with previous studies [37, 38, 39, 40], the consequences of these wrong assumptions and bad communication practices for refugee patients included confusion and missed appointments. For healthcare professionals working with refugees, these communication challenges add to their workload and place an extra burden on them. Healthcare professionals, especially nurses, spend several hours doing ‘behind the scenes’ tasks ranging from simple tasks of doing paperwork to more complex tasks such as helping refugee patients to understand their appointment messages, looking for the meaning of the abbreviations in their clients' appointment reminder messages and sourcing for more information/details about their clients' appointments. It was clear from this study that these tasks are seen as ‘charity work’ and ‘unpaid work’, with considerable additional burden on reception staff, limited numbers of nursing resources and GPs' caseloads already at full capacity. These findings support previous empirical evidence in Australia [30], New Zealand [27] and a systematic review in high‐income countries [28].

These findings of poor practices, such as incorrect assumptions and the use of abbreviations, in health appointments and reminder messages indicate the reliance of health services and professionals on computer systems for communicating health information, such as appointment alerts/reminders. The findings also call for human intervention or oversight of the information generated by computer systems for the purposes of targeting, tailoring and cultural responsiveness [33]. Ultimately, our findings relate to organisational health literacy, which requires services and providers to understand patients' cultural backgrounds, identities and beliefs [9, 41].

For instance, using abbreviations in appointment reminders suggests a lack of organisational health literacy. Instead of services tailoring information to the abilities of refugee patients, the burden is now placed on patients to understand the meaning of the information services share with them [9]. Health providers and administrators must check for understanding of their messages and offer tailored communication in the language of the recipient. By taking a health‐literate approach, health services and professionals, including administrative staff, would understand that appointment communication is not just about sending information to people and assuming they will understand but about making sure the information is easy to read, understandable and tailored to the language.

The findings of this study also align with those reported in other studies outlining the challenges of getting hospital and specialist staff to use interpreters with non‐English‐speaking patients [42, 43]. The findings highlight that most hospital and specialist staff feel reluctant to see refugee patients who do not speak English, as they are not willing to use interpreters. In line with previous studies, providers' unwillingness to use interpreters was attributed to limited consultation time and inadequate knowledge and experience in using interpreters during clinical care [44, 45]. Instances where providers refuse to see refugee patients who are not able to speak English put pressure on healthcare professionals to find practitioners who will accept such patients. Through our study, we highlight that healthcare professionals who face the challenge of finding practitioners willing to use interpreters with refugee patients devise strategies, such as advocating for their clients' right to interpreters. Some of the healthcare professionals also talk to specialists to try to ‘convince’ them to use interpreters with their clients. This advocacy role of healthcare professionals has been reported in an earlier study [24]. Our study recommends that more education on patients' health interpreter rights is needed. Training on interpreter use is also needed to help providers become more knowledgeable.

The findings of our study also support earlier research describing the importance of cross‐cultural interactions and cultural sensitivity in refugee healthcare, including respecting refugees' cultural beliefs and practices, taking interest in refugees' background, language and culture and adopting a compassionate and empathetic approach to care [9, 24, 27, 28]. Nevertheless, participants ensuring that refugee patients receive culturally appropriate services from culturally competent and sensitive specialists are not without challenges. Refugee patients' gender preferences regarding providers present difficulties to GPs in this study when referring them to specialists. Also, healthcare professionals receive complaints of perceived culturally insensitive attitudes of specialists from their refugee clients, with some patients refusing to see such providers again.

The gender preferences and perceived cultural incompetence of specialists place a further demand on healthcare providers, especially GPs, when referring their clients. In some cases, nurses have to attend appointments with their refugee clients for them to feel safe and comfortable, supporting previous studies on healthcare access of refugees [30, 46, 47, 48]. Our findings suggest that bicultural liaison workers can be instituted in refugee health services to accompany newly arrived refugees to their appointments, reducing nurses' workloads. More cultural competence training should also be provided to specialists working with refugee patients to promote access and adherence.

Our findings also echo those of previous research that suggests that primary healthcare professionals' difficulties in referring refugees to appropriate specialist hospital care can be accentuated when they find it difficult to navigate complex health systems themselves due to the siloed nature of refugee services [27, 49]. Participants from our study identified a disconnect between services, leading to feelings of isolation and frustration. Critical reflections are needed on service integration to support refugee health services with strong support networks, ensuring that healthcare professionals feel connected and empowered to address the needs of refugees.

Our study findings also align with evidence from previous Australian and New Zealand studies indicating that primary healthcare providers working with refugees are passionate and committed to their work [27, 30]. Ogunsiji et al. [30] noted that this aspect of providers' experience working with refugees is not adequately echoed in the Australian literature on the primary healthcare workforce in refugee health. Our study participants shared their passion and commitment to helping refugees access hospital and specialist care. Their passion and dedication were shaped by and grounded in their understanding of life as a refugee, equity and fairness, personal conviction and ethics and human rights. Though not explicitly mentioned in the interviews, it is possible that clinicians' passion and commitment are also influenced by their lived experience. They stated that they love and enjoy and even see it as a privilege to provide support to refugees to access services. For instance, some participants felt gratified helping their clients succeed in having a child through in vitro fertilisation. This enjoyment of the participants and the role their passion and commitment play in helping refugees to access hospital and tertiary services have been reported in other studies [30, 50].

However, this study's findings also support those of Kai et al. [51] and Richard et al. [27], who reported considerable uncertainty that healthcare providers may experience when working with refugees, with a potential for professional disempowerment. Our participants felt uncertain about their capacity to continue showing passion and commitment in helping refugees access the hospital and the tertiary‐level care they need. This uncertainty was due to the continued challenges and frustrations they face, as supported by a previous systematic review in high‐income countries [28].

### 4.1 Strengths and Limitations of the Study

This study purposively recruited a sample that was diverse in terms of the characteristics of the participants. These providers were recruited from specialised refugee health services in three Australian states, indicating the representativeness of the findings for these states. The health professionals interviewed had a broad range of experience working with refugees, which enriched the data.

Despite these strengths, this study also has some limitations and the findings should be interpreted in light of these weaknesses. This study included professionals working in specialised refugee health services. Thus, the generalisability of our findings cannot be extended to general practices in the mainstream services. The findings also only reflect healthcare professionals' views and we therefore need to seek the perspectives of refugees in future work to bring those viewpoints into dialogue. This study was conducted during the COVID‐19 pandemic and healthcare professionals were busy and under pressure due to high caseloads. This is likely to impact the length of the interviews.

## 5 Conclusion

This qualitative study provides new insights into refugee healthcare delivery, including the challenges GPs and nurses in specialised refugee health services face in facilitating appointments and referrals to specialist hospital services. The findings call for system improvements—the integration of primary and tertiary care models and responses for refugees.

## Funding

The authors have nothing to report.

## Ethics Statement

The South Western Sydney Local Health District Human Research Ethics Committee approved the research (Approval Number: 2021/ETH11161).

## Consent

Informed consent was obtained from all subjects who participated in this study. Before participating in this study, a participant information sheet detailing this study was provided and written consent was obtained from participants. There was no incentive given to participate in this study.

## Conflicts of Interest

The authors declare no conflicts of interest.

## Data Availability

The data that support the findings of this study are available on request from the corresponding author. The data are not publicly available due to privacy or ethical restrictions.
